# Maryland State Veterinary Medical Society

**Published:** 1901-05

**Authors:** William H. Martenet

**Affiliations:** Secretary


Secretary.

### MARYLAND STATE VETERINARY MEDICAL SOCIETY.

AT a special meeting held on the evening of March 7, 1901, the following resolutions were adopted:

WHEREAS, The untiring zeal and energy of our lately deceased associate and friend, Dr. Albert W. Clement, as pertaining to professional matters in general, were so well known to the veterinary and medical professions that it needs no eulogy to express the esteem in which he was universally held; and

WHEREAS, It has pleased Almighty God to call away our lamented colleague in the pride of youth and at the zenith of his professional career; and

WHEREAS, The Maryland State Veterinary Medical Society keenly realize in his death the loss of one of the most active and useful members and

WHEREAS, Our duties as creatures leads us to the Creator's all-wise dispensation; and

WHEREAS, It is our duty and privilege to tender our deep commiseration to the afflicted; therefore, be it

*Resolved*, That the sincere sympathy of this Society is extended to the bereaved widow and family; and

*Resolved*, That an engrossed copy of these resolutions be tendered the widow; that a copy be spread upon the minute-book, and that a copy be inserted in a daily paper and the journals of the profession.

WILLIAM H. MARTENET, D.V.S.,
Secretary.

